# Improved Viscoelastic Numerical Simulation and In Situ Dynamic FBG Sensing of Interfacial Curing Stress Concentration in Epoxy Insulation Materials

**DOI:** 10.3390/polym18101232

**Published:** 2026-05-18

**Authors:** Zhen Li, Zhiyun Han, Xinkai Zhang, Yizhou Xu, Liang Zou, Kejie Huang, Hanwen Ren

**Affiliations:** 1 School of Electrical Engineering, Shandong University, Jinan 250061, China; 202420753@mail.sdu.edu.cn (Z.L.); hanzhiyun@sdu.edu.cn (Z.H.); xinkaizhang@mail.sdu.edu.cn (X.Z.); 202200190163@mail.sdu.edu.cn (Y.X.); 2 State Key Laboratory of HVDC, Electric Power Research Institute, CSG, Guangzhou 510663, China; huangkj@csg.cn; 3 State Key Laboratory of Alternate Electrical Power System with Renewable Energy Sources, North China Electric Power University, Beijing 102206, China; rhwncepu@ncepu.edu.cn

**Keywords:** epoxy resin, curing stress, numerical simulation, Fiber Bragg Grating (FBG)

## Abstract

Interfacial stress concentration induced by curing shrinkage during the manufacturing of epoxy resin is a primary trigger for micro-nano defect formation and electrical performance degradation in power equipment. To address the computational complexity of traditional viscoelastic models and the thermoelastic behavior wherein the stiffness of the epoxy resin varies with temperature during curing, this paper proposes an improved viscoelastic constitutive model incorporating a thermo-elastic factor. By coupling curing kinetics, heat conduction, chemical shrinkage, and mechanical effects, a multi-physics simulation framework is constructed to describe the complete epoxy curing process, thereby revealing the spatiotemporal evolution of curing stress deformation. To verify the model’s accuracy, an in situ monitoring system based on Fiber Bragg Grating (FBG) sensors was established. A temperature compensation method was utilized to effectively decouple temperature and stress within the complex exothermic curing environment. This study reveals a significant strain gradient effect during the resin curing process. Experimental measurements indicate strains of 21,609 με and 5800 με at the interface and surface, respectively, with numerical simulations exhibiting high agreement with the experimental data. This research not only provides an efficient simulation approach for predicting curing stress but also offers a theoretical basis for the crack-resistant structural design of high-performance epoxy-based power equipment.

## 1. Introduction

In key power equipment such as dry-type reactors, dry-type transformers, and high-voltage bushings, the main insulation is rarely a single medium; rather, it is a composite epoxy insulation system composed of epoxy resin and other materials [1,2]. During the high-temperature curing process of this epoxy system, the mismatch in mechanical, electrical, and thermal properties, combined with inadequate compatibility between the epoxy resin and the other materials, leads to curing stress concentration at the interface. This stress concentration induces micro- and nano-scale defects such as cracks and voids. Partial discharges primarily initiate at these defects and eventually cause insulation failure, which has become a bottleneck restricting the development of power transmission equipment toward ultra-high voltage (UHV) and long-distance transmission [3,4]. Therefore, there is an urgent need to investigate the formation mechanisms and evolution characteristics of interfacial residual stresses that are ubiquitous in the epoxy insulation of power equipment.

Epoxy systems typically employ a multi-stage high-temperature curing process. During manufacturing, curing stresses inevitably arise due to the mismatch in the coefficients of thermal expansion (CTE) among the materials. Its formation and evolution are governed by the complex interplay among external environmental conditions, curing processes, and intrinsic material properties [5]. In the manufacturing stage, the processing environment plays a critical role in stress evolution. Previous studies investigating the effect of the curing environment on stresses in 3D four-directional composite epoxy materials demonstrated that while elevating the curing temperature shortens the curing time, it exacerbates internal residual stress and curing deformation [6]. The cooling rate during the cooling phase also influences curing stress; as the cooling rate increases, curing deformation initially intensifies and subsequently diminishes [7]. Beyond the curing temperature, mold constraints during processing exert external stresses on the epoxy resin, leading to insulation deformation [8]. However, existing studies have predominantly focused on external environmental factors during epoxy curing, often neglecting the evolution of the internal properties of the material itself. Thermosetting resins exhibit pronounced viscoelastic behavior in high-temperature curing environments. The mechanical properties of the epoxy resin, particularly its elastic modulus, vary significantly with the curing conditions and the degree of cure (DOC), undergoing an abrupt transition during the state changes of the material. Furthermore, stress creep and relaxation phenomena occur throughout the curing process, which fundamentally alters the internal stress distribution within the epoxy resin [9].

Finite element (FE) simulation provides an effective approach for the complex computation of curing stresses. The curing of epoxy resin is a multi-physics coupling problem encompassing heat conduction with a nonlinear heat source, cure kinetics, curing mechanics, and fluid dynamics [10], which is typically solved using a sequential coupling method. To resolve the stress–strain field, establishing the curing mechanical constitutive equation of the material—which characterizes the stress–strain–time relationship—is a crucial prerequisite. In early studies on curing residual stresses in composites, material properties were generally simplified as constants, disregarding the effects of DOC, temperature, and other environmental factors on the stress field distribution. However, the properties of epoxy resin, such as the glass transition temperature (*T*_g_) and elastic modulus, are highly dependent on these factors. To account for the contribution of dynamically evolving material properties to curing stress, researchers proposed the Cure Hardening Instantaneously Linear Elastic (CHILE) model based on linear elasticity [11]. To capture viscoelastic characteristics such as stress relaxation and creep, Zocher et al. developed a viscoelastic constitutive model utilizing the Generalized Maxwell model combined with Prony series to represent the relaxation stiffness of the composites [12]. To address the drawback that traditional viscoelastic models require extensive experimental testing to obtain performance parameters (e.g., relaxation times and shift factors), Svanberg et al. proposed a path-dependent simplified viscoelastic constitutive model [13]. Nevertheless, these models inherently assume that the rubbery and glassy stiffnesses of the epoxy resin remain constant, thereby neglecting the material’s thermoelastic characteristics.

In this paper, a multi-physics coupled model incorporating cure kinetics, heat conduction, and curing deformation is first analyzed, and a stiffness factor is introduced to simulate the evolution of physical fields during the epoxy resin curing process. Based on in situ measurement experiments utilizing Fiber Bragg Grating (FBG) sensors, the evolution of residual strain and temperature variations during the curing process was measured in real time. The mechanisms behind interfacial stress concentration were consequently verified and analyzed. This study provides a theoretical foundation for understanding stress formation mechanisms in power equipment, conducting interface-induced failure analysis, and optimizing structural designs.

## 2. Improvement of the Curing Stress Calculation Model for Epoxy Resin Based on the Thermoelastic Stiffness Factor

### 2.1. Generation Mechanism of Curing Stress in Epoxy Resin

**Figure 1:**
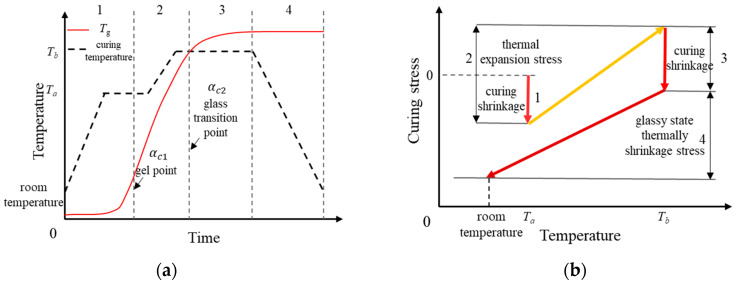
Schematic diagram of the glass transition temperature and curing stress evolution of epoxy resin with respect to temperature and time under a step-curing process: (**a**) material characteristics of epoxy resin; (**b**) evolution characteristics of curing stress.

The curing process of epoxy resin used for power equipment insulation is predominantly a two-stage high-temperature step-curing process. The typical curing stages are illustrated in Figure 1. During the curing process, the thermosetting resin undergoes state transitions from a “viscous flow state” to a “rubbery state” and finally to a “glassy state”, accompanied by corresponding changes in its physicochemical properties.

(1) Flow stage (also known as the pre-gelation stage): In this stage, the resin exhibits a low viscosity and behaves as a liquid (viscous flow state). Under vacuum pressure, air within the epoxy resin layer is evacuated. As the temperature rises, the degree of cure (DOC) of the resin increases, leading to higher viscosity and gradually reduced fluidity until flow ceases at the gel point. Although curing shrinkage and thermal expansion occur during this stage, the liquid state (viscous flow state) of the resin results in negligible mechanical transfer capabilities. Since the shear modulus is nearly zero, any strain induced by volumetric changes is instantaneously relaxed through the free flow of the fluid, thereby preventing the accumulation of macroscopic residual stress within the material. Consequently, the curing stress of the epoxy resin does not exhibit significant variation prior to the gel point. However, some studies indicate that considerable interaction forces may exist between the mold and the resin material, resulting in significant stress prior to the gel point of the epoxy material [14].
(2) Gelation-vitrification stage: The processing temperature in this stage is higher than the glass transition temperature (*T*_g_) of the resin, and the composite material is in a rubbery state. As the temperature continues to rise, the curing reaction accelerates, and the DOC deepens. This stage is the primary interval for the curing reaction. Notably, near the vitrification point, the viscoelasticity of the resin becomes pronounced, and the majority of the curing shrinkage occurs during this period. Due to the low modulus of the resin, the curing shrinkage strain and thermal expansion generated in this stage do not induce significant curing stress, but they substantially affect the material deformation after curing [15].
(3) Post-vitrification holding stage: Vitrification is reached when the *T*_g_ of the resin equals the processing temperature. Subsequently, as *T*_g_ further increases and exceeds the processing temperature, the composite enters the glassy state. During this stage, the change in the DOC is minimal, and the curing shrinkage strain is negligible; therefore, its contribution to the curing stress can be ignored [16].
(4) Cooling stage: In this stage, the material is in the glassy state, and the curing reaction of the resin is essentially complete. Due to the mismatch in the coefficient of thermal expansion (CTE) between the mold and the component, as well as among different layers, the cooling process contributes significantly to the formation of residual curing stress [17].

### 2.2. Cure Kinetics and Mechanical Constitutive Models of Epoxy Resin

Numerical simulation is currently the most widely utilized method for predicting curing deformation and stress. It primarily focuses on calculating the physicochemical processes of the epoxy resin across three stages: the viscous flow state, rubbery state, and glassy state. The numerical model for simulating epoxy resin comprises three modules: the heat conduction-curing module, the flow compaction module, and the stress-deformation module. In this study, E51 epoxy resin was selected as the research material, and its cure kinetics are expressed by the following equation:

(1) $$\left\{\begin{array}{l} \frac{d\alpha }{dt}=\frac{(K_{1}+K_{2}\alpha^{m}){\left(1-\alpha \right)}^{n}}{1+e^{D(\alpha -\alpha_{c})}}\\ K_{1}=A_{1}e^{-\Delta E_{1}/RT}\\ K_{2}=A_{2}e^{-\Delta E_{2}/RT} \end{array}\right.$$

where α is the degree of cure (DOC), *t* is the curing time, and dα/d*t* is the curing rate; *K*_1_ and *K*_2_ are the reaction rate constants, *A*_1_ and *A*_2_ are the pre-exponential factors, Δ*E* is the activation energy, *R* is the universal gas constant, and *T* is the absolute temperature; m and n are the reaction order constants; and *D* is the diffusion factor.

The curing exothermic equation of the epoxy resin is expressed as:

(2) $$q=\rho \Delta H\frac{d\alpha }{dt}$$

where *q* is the heat flux of the curing exotherm, *ρ* is the density of the epoxy resin, and Δ*H* is the total heat of reaction. Assuming that the density, specific heat capacity, and thermal conductivity of the epoxy resin are linearly correlated with the DOC, they are respectively expressed by the following equations:

(3) $$\rho =\rho_{0}(1-\alpha )+\rho_{1}\alpha$$

(4) $$C_{p}=C_{p0}(1-\alpha )+C_{p1}\alpha$$

(5) $$k=k_{0}(1-\alpha )+k_{1}\alpha$$

The core assumption of the Cure Hardening Instantaneously Linear Elastic (CHILE) model is that, at any instant during the curing process, the stress–strain relationship within the resin and the composite conforms to linear elastic behavior, while the instantaneous modulus of the resin depends on the current temperature and DOC [8]. The dependence of the instantaneous modulus on these variables is typically provided by empirical formulas. Current mainstream models include CHILE(*α*) and CHILE(*T*_g_). In the CHILE(*α*) model, the instantaneous modulus of the epoxy resin is given by:

(6) $$E_{r=}\left\{\begin{array}{l} E_{r}^{0},\;\alpha <\alpha_{c1}\\ \left(1-\alpha_{\mathrm{mod}}\right)E_{r}^{0}+\alpha_{\mathrm{mod}}E_{r}^{0}\;+\\ \gamma_{r}\alpha_{\mathrm{mod}}\left(1-\alpha_{\mathrm{mod}}\right)\left(E_{r}^{\infty }-E_{r}^{0}\right),\;\alpha_{c1}\leq \alpha \leq \alpha_{c2}\\ E_{r}^{\infty },\;\alpha >\alpha_{c2} \end{array}\right.$$

(7) $$\alpha_{\mathrm{mod}}=\frac{\alpha -\alpha_{c1}}{\alpha_{c2}-\alpha_{c1}}$$

where α_c1_ and α_c2_ denote the boundary values of the DOC, corresponding to the DOC at the vitrification point and gel point, respectively; $E_{r}^{0}$ and $E_{r}^{\infty }$ represent the rubbery modulus and glassy modulus of the epoxy resin, respectively; *γ*_r_ is a factor representing the two competing mechanisms of stress relaxation and cure hardening. This constitutive model divides the evolution of the resin modulus into three stages, with the modulus remaining constant in the first and third stages. In the first stage, the DOC is low, and the modulus is extremely small. In the second stage, the modulus increases significantly with the increasing DOC, and curing shrinkage primarily occurs during this phase. In the third stage, as curing nears completion, the resin modulus remains constant, and curing shrinkage ceases.

However, when the curing temperature approaches the *T*_g_, the modulus of the material undergoes a substantial change—a phenomenon that the CHILE(α) model fails to capture. To address this limitation, Johnston et al. proposed an improved CHILE model, namely CHILE(*T*_g_), in which material properties are correlated with *T*_g_ [18]. The modulus of the resin during the curing process is:

(8) $$E_{r=}\left\{\begin{array}{l} E_{r}^{0},\;T<T_{c1}\\ E_{r}^{0}+\frac{T-T_{c1}}{T_{c2}-T_{c1}}\left(E_{r}^{\infty }-E_{r}^{0}\right),\;T_{c1}\leq T\leq T_{c2}\\ E_{r}^{\infty },\;T>T_{c2} \end{array}\right.$$

where *T*_c1_ and *T*_c2_ represent the boundary values of *T*. As the relationship between *T*_g_ and α is crucial, the DiBenedetto equation [16] is widely employed to describe it:

(9) $$\frac{T_{g}-T_{g0}}{T_{g\infty }-T_{g0}}=\frac{\lambda \alpha }{1-(1-\lambda )\alpha }$$

where *T*_g0_ is the glass transition temperature at a DOC of 0, *T*_g∞_ is the glass transition temperature of the fully cured epoxy resin, and *λ* is a material constant, typically taken as 0.43.

Fundamentally, both the CHILE(α) and CHILE(*T*_g_) models are linear elastic constitutive models. Given that epoxy resin exhibits pronounced viscoelastic properties at high temperatures, these models are inadequate for reflecting characteristics such as stress relaxation or creep.

### 2.3. Improvement of the Curing Mechanical Constitutive Equation of Epoxy Resin Based on Thermoelastic Stiffness

To circumvent the complex performance parameters required by traditional viscoelastic models, Svanberg et al. proposed a path-dependent simplified viscoelastic constitutive model [19], wherein rate-dependent material parameters are replaced by state-dependent ones. In the path-dependent model, the material properties of the resin and the composite remain constant in the glassy and rubbery states, undergoing a step change when the temperature reaches *T*_g_. The variation of the resin properties in the path-dependent model is expressed as:

(10) $$E_{r}=\left\{\begin{array}{l} E_{r}^{0},\;T\leq T_{g}(\alpha )\\ E_{r}^{\infty },\;T>T_{g}(\alpha ) \end{array}\right.$$

The corresponding incremental equation of the constitutive model is:

(11) $$\Delta \sigma_{i}=\left\{\begin{array}{l} C_{ij}^{0}\Delta \varepsilon_{j},\;T\leq T_{g}(\alpha )\\ C_{ij}^{\infty }\Delta \varepsilon_{i}-S_{i},\;T>T_{g}(\alpha ) \end{array}\right.$$

As indicated by the equations, the path-dependent model hypothesizes that material properties experience a step change at the vitrification point. However, conventional viscoelastic models inherently assume that the equilibrium stiffness (rubbery stiffness) and initial stiffness (glassy stiffness) of the epoxy resin are constants. Conversely, experimental evidence demonstrates that both equilibrium and initial stiffnesses are temperature dependent [20].

When the epoxy resin is in the rubbery state, the material’s resistance to deformation is no longer primarily provided by intermolecular forces but rather driven by the conformational entropy of the molecular chains. According to the Kinetic Theory of Rubber Elasticity, the higher the thermal energy of the molecular chains, the stronger their tendency to maintain a coiled state through thermal motion. Consequently, the modulus is directly proportional to the temperature, expressed as:

(12) $$E_{r}(T)=E_{r}^{0}+k_{r}T$$

where *k*_r_ is the rubbery temperature softening coefficient, taken as 0.05 MPa/K.

In the glassy state, the molecular chain segments are frozen, and the material behaves as an elastic solid. The stiffness at this stage is primarily determined by intermolecular van der Waals forces and bonding energy (enthalpy-driven). As the temperature rises, molecular thermal vibrations intensify, and the material’s thermal expansion leads to an increase in “free volume”. This expands the intermolecular distance and weakens the interaction forces, macroscopically manifesting as a gradual decline in stiffness. This phenomenon is typically described using a linear empirical decay equation:

(13) $$E_{g}(T)=E_{r}^{\infty }-k_{g}(T-T_{\mathrm{ref}})$$

where $E_{r}^{\infty }$ is the glassy modulus at the reference temperature *T*_ref_, and *k*_g_ is the glassy temperature softening coefficient, taken as 10 MPa/K. Thus, Equations (12) and (13) are substituted into the constitutive model.

## 3. Multi-Physics Simulation of the Epoxy Resin Curing Process

### 3.1. Parameter Settings

Based on the aforementioned curing stress constitutive model, the finite element method (FEM) was employed to simulate the thermal treatment and curing processes of a simplified coaxial epoxy resin structure. The material property settings are detailed in Table 1 and Table 2.

The 3D simulation model was established as a simplified 1:1 replica of an actual 35 kV single-encapsulation dry-type reactor. Physically, after the metal conductor windings are coiled, they are encapsulated by epoxy resin to form a cylindrical structure. Given that the dry-type reactor possesses a typical axisymmetric configuration—where both the windings and the insulation encapsulation exhibit geometric and material uniformity along the circumferential direction—the 3D structure is reasonably simplified into a 2D axisymmetric cross-sectional model for analysis. The specific geometric parameters of the simplified model are detailed in Table 3.

**Figure 2:**
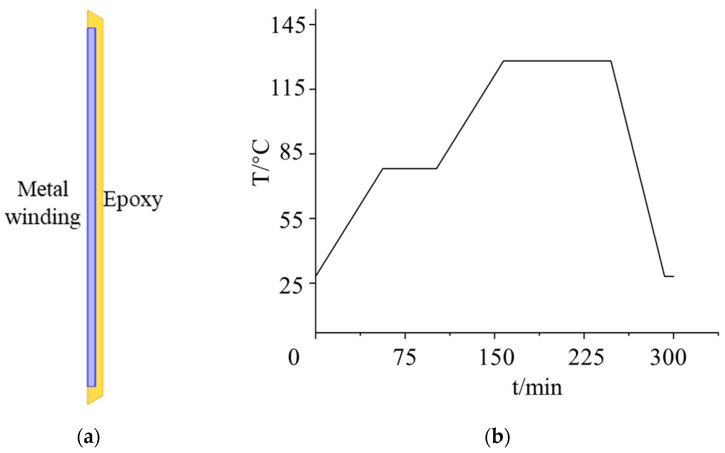
Simulation settings. (**a**) Geometric model; (**b**) curing temperature profile.

The standard curing procedure for E51 epoxy resin involves a typical two-stage high-temperature step-curing process, as illustrated in Figure 1. The geometric model and curing temperature profile is depicted in Figure 2. Specifically, the epoxy resin was initially cured at 80 °C for one hour, subsequently heated to 130 °C for a two-hour curing phase, and finally cooled to room temperature prior to demolding.

### 3.2. Simulation Results and Analysis of Temperature and Degree of Cure

**Figure 3:**
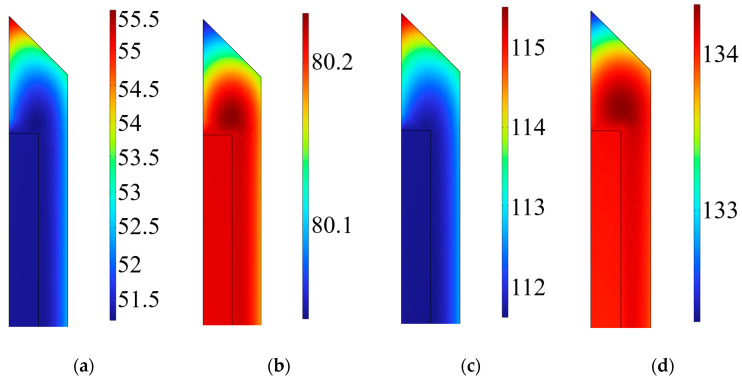
Temperature variation of the epoxy resin. (**a**) t = 50 min; (**b**) t = 100 min; (**c**) t = 150 min; (**d**) t = 200 min.

**Figure 4:**
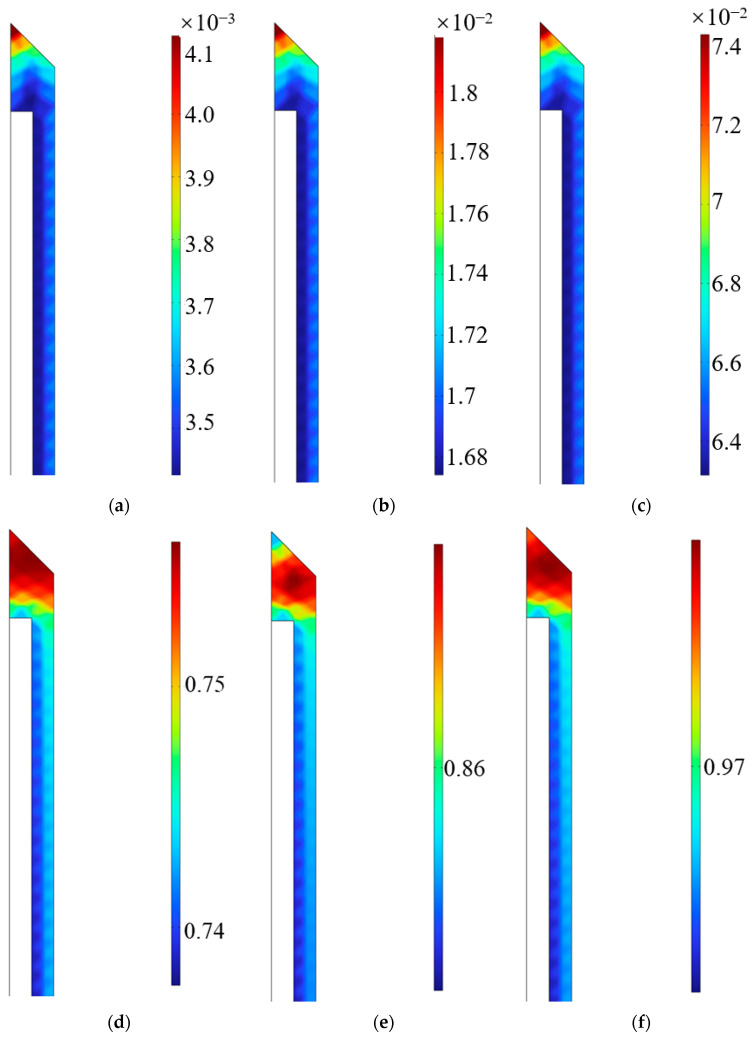
DOC Variation of the epoxy resin. (**a**) t = 50 min; (**b**) t = 100 min; (**c**) t = 150 min; (**d**) t = 200 min; (**e**) t = 250 min; (**f**) t = 300 min.

Firstly, the temperature evolution within the epoxy model was investigated, as shown in Figure 3. Because the heat transfer path exhibits a spatial gradient from the exterior to the interior, and the curing cross-linking of the epoxy resin is inherently a highly exothermic chemical polymerization process, significant thermo-chemical coupling effects are induced, leading to asynchronous local curing. As the ambient temperature rises, the exothermic rate of the internal reaction intensifies. The internally generated heat of reaction cannot diffuse to the outer boundaries promptly, resulting in a heat accumulation phenomenon. The DOC contours of the epoxy resin at various times were extracted for analysis, as depicted in Figure 4.

**Figure 5:**
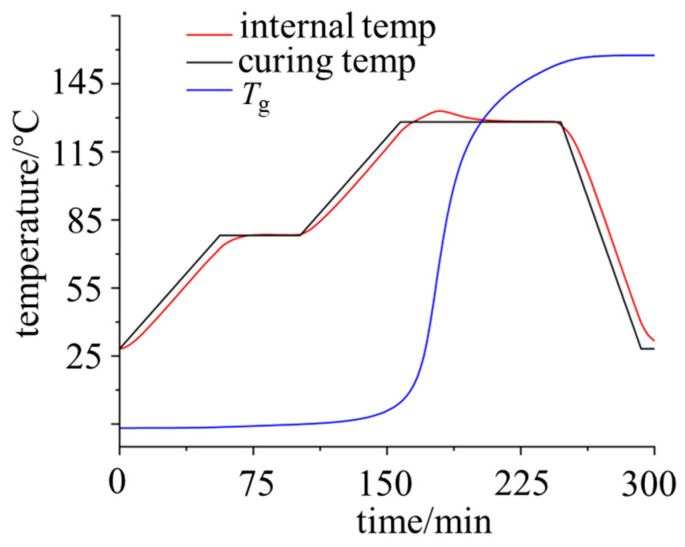
Curves of internal temperature and glass transition temperature of the epoxy.

The internal temperature variation of the epoxy was further compared with the designated curing temperature profile, as illustrated in Figure 5. When the external temperature exceeded the gelation temperature of the epoxy resin, the material began to cure. At 175 min, during the second heating stage, the maximum internal temperature of the epoxy reached approximately 140 °C, which was significantly higher than the external temperature, indicating that the exothermic heat of the curing reaction had reached its peak. Combined with Figure 3d, this demonstrates that heat accumulation occurs within the epoxy resin during curing, which further facilitates the complete curing of the material.

Furthermore, as observed in Figure 5, the temperature variation at the interface lagged behind that at the free surface during the curing process, resulting in delayed epoxy curing at the interface. Due to the differences in heat capacity and thermal diffusivity between the epoxy and the mold at the interface, the thermal response rate of the interface lagged significantly behind that of the free surface. This thermodynamic time-phase difference directly caused an overall delay in the curing process of the epoxy resin at the interface. This finding also indirectly elucidates the physical mechanism behind the propensity for residual stress concentration at the interface in subsequent stages.

### 3.3. Analysis of Curing Stress Simulation Results

**Figure 6:**
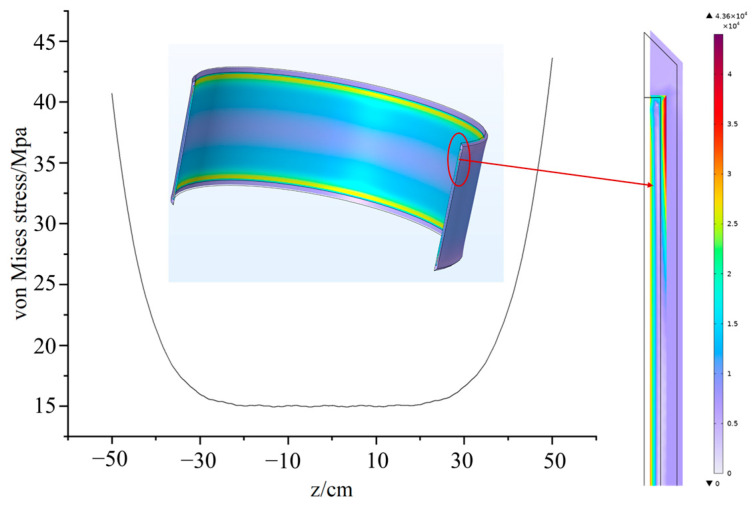
Stress distribution of the epoxy resin model.

Figure 6 illustrates the stress characteristics at the interface at the end of the cooling stage for the epoxy resin. The curve illustrates the interfacial stress distribution of the epoxy resin along the *z*-axis, the stress distribution exhibits a “U” shape, with abrupt stress transitions occurring at the interface edges. The stresses at the top and bottom edges reach 40.704 MPa and 43.617 MPa, respectively. The right panel illustrates the stress distribution contour for the upper section of the encapsulation. The stress gradually increases as it approaches the interface, indicating a pronounced interfacial concentration effect. The maximum stress value emerges at the top edge of the interface, rendering this location a weak point in terms of mechanical strength. At the free edges or geometrically discontinuous regions of the structure, the deformation compatibility conditions of the material change drastically. This leads to an extreme local concentration of shear and normal tensile stresses, thereby forming a steep stress gradient.

**Figure 7:**
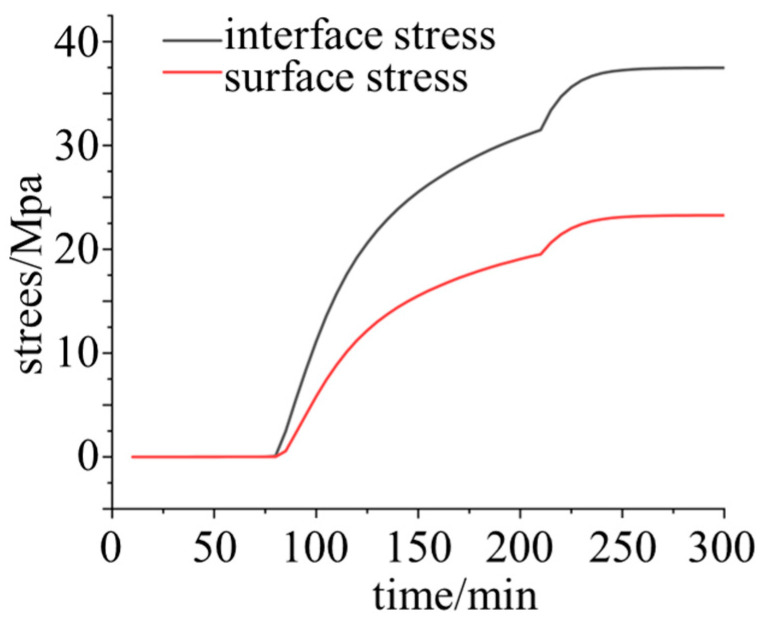
Comparison of the curing stress evolution between the interface and the surface of the epoxy resin.

The stress evolution characteristics at the top edge boundary and the surface of the epoxy resin are depicted in Figure 7. Once the epoxy resin transitions past the rubbery state, the cross-linking density increases sharply, accompanied by volumetric shrinkage (curing shrinkage) induced by chemical cross-linking. Under structural constraints, the material cannot deform freely, triggering a rapid surge in internal curing stress within this interval. After 250 min, the DOC approaches saturation, and the stress curve gradually flattens and converges. Under the strong constraint of the rigid physical boundary of the metallic material, the interfacial curing stress exhibits a high degree of concentration. Furthermore, the mismatch in the CTE between the resin and the metal further exacerbates the stress concentration phenomenon at the interface.

## 4. Strain Detection in Epoxy Resin Based on FBG Sensors

### 4.1. Measurement Principle of FBG

The operational principle of an FBG sensor relies on the periodic variation of the refractive index within the fiber core to reflect a specific wavelength of light (i.e., the Bragg wavelength). Any external ambient temperature variation or mechanical strain disrupts this physical periodicity and alters the effective refractive index of the optical fiber, consequently resulting in a measurable shift in the reflected wavelength [21]. Specifically, variations in strain and temperature induce the photoelastic effect and the thermo-optic effect within the fiber. The reflected wavelength is a function of the effective refractive index of the optical fiber and the grating period [22]. FBG sensors are characterized by rapid response, real-time monitoring capabilities, and high sensitivity to external perturbations. The shift in the reflected wavelength exhibits a linear relationship with the axial strain and the temperature variation surrounding the grating, expressed as follows:

(14) $$\frac{\Delta \lambda_{B}}{\lambda_{B}}=(1-P_{e})\varepsilon +(\alpha_{f}+\xi )\Delta T$$

where λ_B_ and Δλ_B_ are the Bragg center wavelength of the FBG sensor and its corresponding shift, respectively; *P*_e_ is the effective photoelastic coefficient (strain sensitivity coefficient); ε represents the axial strain; α*_f_* and ξ denote the thermal expansion coefficient and thermo-optic coefficient (temperature sensitivity coefficients), respectively; and Δ*T* is the temperature variation.

### 4.2. In Situ Measurement Experiment of the Curing Process

The formulation system employed in this study was referenced from the actual systems used in dry-type reactors. Bisphenol-A epoxy resin E51, with an epoxy equivalent weight (EEW) of 196 g/mol, was utilized. Methylhexahydrophthalic anhydride (MHHPA) served as the curing agent, and 2,4,6-tris(dimethylaminomethyl)phenol (DMP-30) was selected as the catalyst. Based on the anhydride dosage formula and relevant literature, the mass ratio of the epoxy resin, curing agent, and catalyst was set to 1:0.82:0.02. The inner and outer diameters of the PTFE mold are set to 80 mm and 100 mm, respectively, with the epoxy resin layer having a thickness of 2 mm.

**Figure 8:**
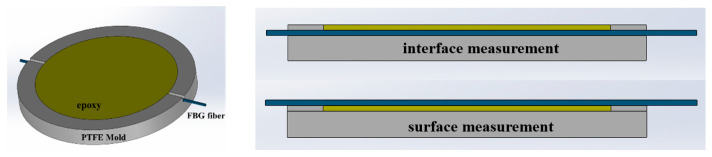
Assembly diagram of the epoxy resin sample and the mold embedded with FBG sensors.

**Figure 9:**
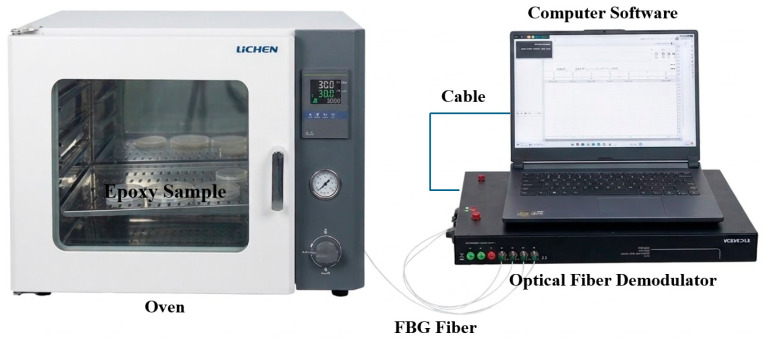
In situ strain/temperature measurement system based on Fiber Bragg Grating (FBG) sensors.

The FBG sensors (manufactured by Wuhan FBTK, Wuhan, China) featured a center wavelength of 1541.997 nm and a grating length of 5 mm. An FBTK-D40 optical interrogator with a wavelength range of 1525–1565 nm was used for signal acquisition. The specific experimental procedures were as follows:

1. The epoxy resin was preheated in a 60 °C water bath for 20 min. Subsequently, the curing agent was added according to the specified ratio, and the mixture was mechanically stirred at 450 rpm at 60 °C for 30 min. The catalyst was then introduced, followed by an additional 30 min of stirring.
2. The stirred epoxy resin matrix was placed in a vacuum drying oven at 60 °C for 30 min to undergo degassing.
3. A pre-slotted mold, previously coated with a release agent, was preheated at 80 °C for 30 min. The degassed epoxy resin was then poured into the mold. Simultaneously, the FBG sensors were embedded at the bottom and top of the mold, respectively. The diameter of the bare FBG optical fiber is approximately 150 μm. Compared to the macroscopic dimensions of the epoxy resin and the PTFE mold (80 mm), the thickness perturbation introduced by the sensor is entirely negligible. Furthermore, to secure the optical fiber at the bottom of the mold, pre-fabricated slots are introduced at both ends of the mold to route the measurement fiber. The fiber is then firmly anchored using an adhesive, ensuring that it is precisely positioned at the physical interface between the epoxy resin and the mold. The assembly diagram is shown in Figure 8.
4. The potted mold was placed into a programmable oven, and the optical fibers were routed out and connected to the interrogator. The curing process proceeded according to the predefined temperature profile. Because FBG sensors are sensitive to both strain and temperature, an independent temperature-sensing optical fiber was placed in the oven to decouple the temperature from the measurement results of the embedded fibers. After curing was complete and the system had cooled to room temperature, the measurement data were collected. The experimental setup is illustrated in Figure 9.

### 4.3. Analysis of Measurement Results

**Figure 10:**
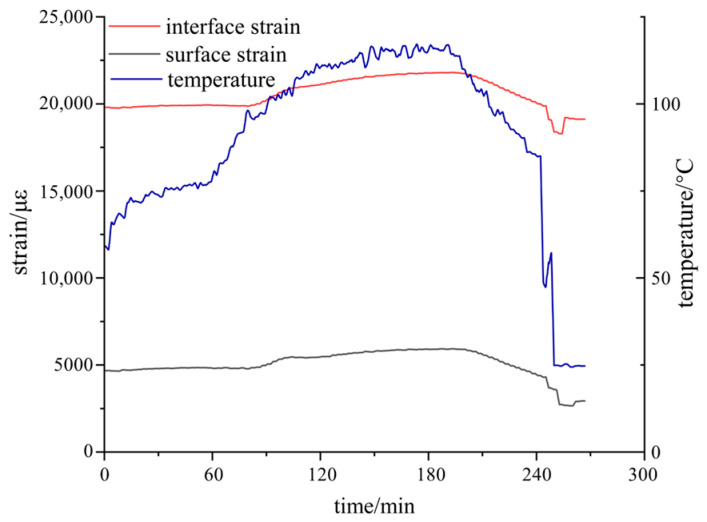
Measurement of curing stress evolution at the interface and surface of the epoxy resin.

The strain measurement results are presented in Figure 10. In the initial stage, the interfacial strain of the epoxy resin sample was maintained at approximately 19,800 με. Subsequently, as the epoxy entered the viscous flow state, the strain increased. With the sharp decrease in temperature, the strain underwent significant relaxation and reduction. After the temperature stabilized, the strain eventually settled at around 19,100 με.

The generation and evolution of stress exhibited a high positive correlation with the curing temperature, indicating that thermodynamics is the core factor driving the evolution of curing stress in the epoxy resin. As the temperature decreased, the material underwent thermal shrinkage. Due to the geometric constraints imposed by the internal structure of the system or the material interfaces, this shrinkage of free volume was restricted, thereby leading to the release or redistribution of internal residual stresses.

A comparison between the interfacial and surface strains reveals that the absolute value of the interfacial stress is substantially higher than that of the surface stress. This phenomenon aligns with the typical mechanical characteristics of multi-layer composite structures during curing and molding. The interface frequently becomes the primary region of stress concentration owing to the mismatch in the coefficients of thermal expansion (CTE) between different materials, coupled with the strong constraint effects induced by chemical bonding or mechanical interlocking. Conversely, the surface region, lacking strong external rigid constraints, exhibits a relatively lower stress level, and the magnitude of stress release is correspondingly smoother.

## 5. Conclusions

To address the interfacial stress concentration issue in the epoxy composite insulation of power equipment during the curing process, this paper proposed an improved viscoelastic numerical simulation method and conducted experimental validation utilizing FBG sensing technology. The main conclusions drawn are as follows:

(1) Breaking through the theoretical limitations of traditional constitutive models, this study innovatively proposes a multi-physics coupled simulation framework incorporating a thermoelastic factor. Traditional linear elastic models fail to accurately characterize the abrupt stiffness transitions of polymer materials during state changes. Therefore, this paper profoundly reveals the underlying coupling mechanisms among curing kinetics, heat conduction, and viscoelastic mechanical behaviors. This framework not only achieves high-fidelity predictions of the spatiotemporal evolution of curing deformation and interfacial stress in epoxy resins but also provides a reliable, novel methodology for analyzing the mechanical responses of composite insulation structures under complex thermal boundaries.
(2) This study quantitatively resolves the stress surge mechanism dominated by thermo-mechanical coupling during the curing process, accurately pinpointing the mechanical weak links of the insulation structures in dry-type cylindrical power equipment. The analysis indicates that the competition between internal exothermic reactions and external heat conduction leads to significant heat accumulation and curing hysteresis effects. Particularly, after the material transitions beyond the rubbery state, the chemical shrinkage dominated by strong structural constraints becomes the core driving force for the explosive growth of stress. Under the combined effects of rigid boundaries and the mismatch in the coefficients of thermal expansion (CTE) of the materials, an extreme stress value of up to 43.617 MPa is generated at the interface. This finding provides explicit theoretical guidance at the mechanistic level for the structural optimization and curing process improvement of high-voltage power equipment, such as dry-type reactors.
(3) The research confirms the central role of thermodynamic evolution in stress redistribution and establishes an engineering application paradigm that combines in situ monitoring with high-fidelity simulation. By utilizing a self-constructed embedded FBG sensing system, the precise in situ decoupling of temperature and strain under complex multi-field environments was successfully achieved. The extreme interfacial strain measured in the experiment (approximately 19,800 με) not only corroborates the accuracy of the proposed multi-physics coupled model but also intuitively exposes the severe risk of interfacial stress concentration in multi-layer composite structures. These research outcomes provide highly promising technical support for the non-destructive evaluation, process design optimization, and long-term operating condition early-warning of large-scale epoxy-cast solid insulation equipment.

## Figures and Tables

**Table 1:** Cure kinetic and thermophysical parameters of E51 epoxy resin.

Parameter	Value
m	2.75
n	3.9
Δ*E*_1_	7.07 × 10^4^ J/mol
Δ*E*_2_	6.71 × 10^4^ J/mol
*A*_1_	9.64 × 10^6^ 1/s
*A*_2_	1.36 × 10^3^ 1/s
Δ*H*	402 J/g
*ρ*_0_	1108.9 kg/m^3^
*ρ*_1_	1168.7 kg/m^3^
*C*_p0_	1763 J/(kg·K)
*C*_p1_	1171 J/(kg·K)
*k*_0_	0.16 W/(m·K)
*k*_1_	0.22 W/(m·K)

**Table 2:** Curing mechanical parameters of E51 epoxy resin.

Parameter	Value
$E_{r}^{0}$	9.5 Mpa
$E_{r}^{\infty }$	9.5 × 10^3^ Mpa
*T*_g0_	−47.78 °C
*T*_g∞_	84.03 °C
α_c1_	1.09 × 10^−5^ 1/K
α_c2_	3.15 × 10^−5^ 1/K

**Table 3:** Geometric parameters of the simulation model.

Parameter	Value (mm)
Winding inner diameter	500
Winding thickness	5
Insulation outer diameter	510
Insulation thickness	5
Encapsulation height	1000
Encapsulation chamfer	10

## Data Availability

The original contributions presented in this study are included in the article. Further inquiries can be directed to the corresponding author.

## References

[1] Lokanathan M., Acharya P.V., Ouroua A., Strank S.M., Hebner R.E., Bahadur V. (2021). Review of Nanocomposite Dielectric Materials with High Thermal Conductivity. Proc. IEEE.

[2] Wu Z., Lin B., Fan J., Zhao J., Zhang Q., Li L. (2022). Effect of Dielectric Relaxation of Epoxy Resin on Dielectric Loss of Medium-Frequency Transformer. IEEE Trans. Dielectr. Electr. Insul..

[3] Jin F.-L., Li X., Park S.-J. (2015). Synthesis and Application of Epoxy Resins: A Review. J. Ind. Eng. Chem..

[4] Shengtao L., Mingru L. (2024). Development of Epoxy Resin with Superior Breakdown Strength: A Review. iEnergy.

[5] Li Q., Weinell C.E., Kiil S. (2022). Curing-Induced Internal Stress in Epoxy Coatings: Effects of Epoxy Binder, Curing Agent, Filler, Initial Solvent Concentration, Curing Temperature, and Relative Humidity. Prog. Org. Coat..

[6] Fu Y., Gao X., Yao X. (2020). Mesoscopic Simulation on Curing Deformation and Residual Stresses of 3D Braided Composites. Compos. Struct..

[7] Miao Y., Li J., Gong Z., Xu J., He K., Peng J., Cui Y. (2013). Study on the Effect of Cure Cycle on the Process Induced Deformation of Cap Shaped Stiffened Composite Panels. Appl. Compos. Mater..

[8] Ledru Y., Bernhart G., Piquet R., Schmidt F., Michel L. (2010). Coupled Visco-Mechanical and Diffusion Void Growth Modelling during Composite Curing. Compos. Sci. Technol..

[9] Gerritzen J., Müller-Pabel M., Müller J., Gröger B., Lorenz N., Hopmann C., Gude M. (2022). Development of a High-Fidelity Framework to Describe the Process-Dependent Viscoelasticity of a Fast-Curing Epoxy Matrix Resin Including Testing, Modelling, Calibration and Validation. Polymers.

[10] Cheng S., Tong Y., Li G., Gao J., Yue T. (2025). An Innovative Semi-Surrogate Model Approach for Numerical Simulation of CO2 Bubble Reaction in Alkanolamine Solutions: Balancing Computational Efficiency and Accuracy. Powder Technol..

[11] Bogettit A., Gillespie J. (1992). Process-Induced Stress and Deformation in Thick-Section Thermoset Composite Laminates. Composites.

[12] Baran I., Cinar K., Ersoy N., Akkerman R., Hattel J. (2017). A Review on the Mechanical Modeling of Composite Manufacturing Processes. Arch. Comput. Methods Eng..

[13] Magnus J., Anders J. (2004). Prediction of Shape Distortions Part I. FE-Implementation of a Path Dependent Constitutive Model. Compos. Part A Appl. Sci. Manuf..

[14] Sorrentino L., Marchetti M., Bellini C., Delfini A., Del Sette F. (2017). Manufacture of High Performance Isogrid Structure by Robotic Filament Winding—ScienceDirect. Compos. Struct..

[15] Ersoy N., Potter K., Wisnom M.R., Clegg M.J. (2005). Development of Spring-in Angle during Cure of a Thermosetting Composite. Compos. Part A Appl. Sci. Manuf..

[16] Wang C., Zhou G., Sun Q., Chen C., Zhang Z., Li H., Peng Z. (2022). Numerical Simulation of the Interfacial Stress between Epoxy Resin and Metal Conductor of Power Equipment during Epoxy Curing. High Volt..

[17] Salomi A., Garstka T., Potter K., Greco K., Maffezzoli A. (2008). Spring-in angle as molding distortion for thermoplastic matrix composite. Compos. Sci. Technol..

[18] Johnston A., Vaziri R., Poursartip A. (2001). A Plane Strain Model for Process-Induced Deformation of Laminated Composite Structures. J. Compos. Mater..

[19] Prasatya P., BMcKenna G., LSimon S. (2001). A Viscoelastic Model for Predicting Isotropic Residual Stresses in Thermosetting Materials: Effects of Processing Parameters. J. Compos. Mater..

[20] Ding A., Li S., Zu L., Wang J. (2015). High temperature behaviour of glass-vinyl ester stack liner. Plast. Rubber Compos..

[21] He Y., Yuan M., Li Q., Tang L., Yang W., Ping Y., He H., Deng B. (2025). Feasibility Study of Mechanical Stress Wave Detection in Power Semiconductor Devices Using Bare FBG Sensors. IEEE Sens. J..

[22] Guo K., Wu H., Liang Y., Su M., Wang H., Chu R., Zhou F., Liu Y. (2025). Highly Birefringent FBG Based on Femtosecond Laser-Induced Cladding Stress Region for Temperature and Strain Decoupling. Photonics.

